# Tumor infiltrating CD8/CD103/TIM-3-expressing lymphocytes in epithelial ovarian cancer co-express CXCL13 and associate with improved survival

**DOI:** 10.3389/fimmu.2022.1031746

**Published:** 2022-10-21

**Authors:** Martijn Vlaming, Vrouyr Bilemjian, Jimena Álvarez Freile, Vinicio Melo, Annechien Plat, Gerwin Huls, Hans W. Nijman, Marco de Bruyn, Edwin Bremer

**Affiliations:** ^1^ Department of Hematology, University of Groningen, University Medical Center Groningen, Groningen, Netherlands; ^2^ Department of Obstetrics & Gynecology, University of Groningen, University Medical Center Groningen, Groningen, Netherlands

**Keywords:** tumor infiltrating lymphocytes, epithelial ovarian cancer, TIM-3, CXCL13, RNA-seq

## Abstract

Reactivation of tumor infiltrating T lymphocytes (TILs) with immune checkpoint inhibitors or co-stimulators has proven to be an effective anti-cancer strategy for a broad range of malignancies. However, epithelial ovarian cancer (EOC) remains largely refractory to current T cell-targeting immunotherapeutics. Therefore, identification of novel immune checkpoint targets and biomarkers with prognostic value for EOC is warranted. Combining multicolor immunofluorescent staining’s with single cell RNA-sequencing analysis, we here identified a TIM-3/CXCL13-positive tissue-resident memory (CD8/CD103-positive) T cell (Trm) population in EOC. Analysis of a cohort of ~175 patients with high-grade serous EOC revealed TIM-3-positive Trm were significantly associated with improved patient survival. As CXCL13-positive CD8-positive T cells have been strongly linked to patient response to anti-PD1 immune checkpoint blockade, combinatorial TIM-3 and PD-1 blockade therapy may be of interest for the (re)activation of anti-cancer immunity in EOC.

## Introduction

For epithelial ovarian cancer (EOC) the infiltration of lymphocytes, particularly T cells, in tumor tissue is associated with a better clinical outcome (1–3), suggesting T cell immunotherapy may be of use. However, although re-activation of immune cells with immune checkpoint inhibitors (ICI) or co-stimulators is effective for a wide range of malignancies (as reviewed in (4–6)), responses in EOC are disappointing. Most notably, objective responses in EOC do not exceed 7% with PD-1 checkpoint therapy (7–9). Accordingly, releasing the PD-1/PDL-1 immune checkpoint brake with ICI´s is not sufficient for re-establishing anti-tumor immunity in EOC. For this reason, identification of novel immune checkpoint targets on TILs as well as biomarkers with prognostic value in EOC remains essential.

Intra-epithelial localization of CD8-positive TILs is associated with improved patient survival in EOC, whereas intratumoral yet stromally located TILs do not associate with survival (10–14). More specifically, survival is associated with a subset of TILs that can be identified by CD103 expression, an αE integrin subunit regarded as a tissue-resident memory T cell marker. This TIL population additionally expresses immunotherapeutic targets, such as PD-1 and CD27, and can possibly be re-activated by a combination of PD-1 checkpoint inhibition and CD27 co-stimulators (12). This subpopulation is defined as highly activated, tumor-specific, and tissue-resident memory T cells that can also express the T cell immunoglobulin and mucin domain 3 (TIM-3) checkpoint receptor (15). In line with this, CD8-positive tumor-reactive T cells in different solid tumors co-express PD-1, LAG-3, CTLA-4 and TIM-3 (16, 17).

Initially, TIM-3 expression was described on IFN-γ producing CD4-positive helper T cells and cytotoxic CD8-positive T cells and reported to regulate macrophage activation (18). On TIL populations, TIM-3 expression is associated with T cell exhaustion, tumor progression, and poor clinical outcome in certain cancers (19–22). Reversely, for other cancers, it can associate with benefit. For instance, TIM-3 expression in TILs of triple-negative breast cancer (TNBC) patients associated with longer recurrence-free and overall survival (23). However, in a meta-analysis study of 3,072 cases from 21 published studies from a range of solid cancers TIM-3 expression on TILs did not associate with overall survival. In contrast, TIM-3 expression on malignant cells did significantly associate with poor overall survival (24). Thus, solely evaluating TIM-3 expression levels within a TIL population is likely not sufficient and a more detailed delineation of subsets of TILs that express TIM-3 is warranted. Particularly, whether such a more relevant TIL population associates with patient survival and may thus be of therapeutic interest.

Interestingly, expression of the chemokine CXCL13 has also been identified in highly exhausted TIM-3-expressing TILs, a cell population that was predictive for both response to ICIs (PD-1 blockade) and survival in non-small cell lung cancer patients and muscle-invasive bladder cancer (MIBC) (25, 26). CXCL13 expression itself associated with prognosis, immune infiltration, and T cell exhaustion in ovarian cancer (27). CXCL13 expression was TGFβ-dependent and mediated B cell recruitment and formation of tertiary lymphoid structures (TLSs) in human tumors (28). Notably, although not directly evaluated in the current study, the presence of TLSs in several human tumors has been linked to improved prognosis and outcome upon immunotherapy (29–31). Further, tumor infiltrated CD8-positive T cells in tumors without TLSs lacked prognostic benefit or even associated with increased risk of disease progression (32, 33).

Here, a cohort of EOC core samples was evaluated for the presence of tumor infiltrating CD8/CD103/TIM-3 triple-positive T cells and subsequently correlated with patient survival. Interestingly, increased tumor infiltration of CD8/CD103/TIM-3 triple-positive cells associated with improved patient survival in EOC, suggesting that CD8/CD103/TIM-3 triple-positive TILs can serve as a prognostic marker for EOC. In line with this finding, a single-cell tumor immune transcriptomic dataset revealed co-expression of TIM-3, CXCL13 and CD103 within the terminally exhausted CD8-positive T cell fraction (pre-defined by using canonical markers and curated gene signatures (34)). Expression of CXCL13 could predominantly be attributed to the CD8/CD103/TIM-3 triple-positive fraction compared to single- and double-positive counterparts in primary EOC samples. Thus, TIM-3 expression on CD8/CD103-double positive TILs may be used as surrogate marker for prognostically favorable CXCL13-positive CD8-positive TILs and may have prognostic value.

## Materials and methods

### Patient selection

Patient selection and construction of the tissue micro-array (TMA) were described previously (35). Briefly, a recoded database was created containing information on clinico-pathological characteristics and follow-up of patients diagnosed with advanced stage HGSOC at the University Medical Center Groningen (Groningen, The Netherlands) and Isala hospital Zwolle (Zwolle, The Netherlands) between January 2008 and January 2017. In total 176 EOC patients were included from participating centers (see **Table 1**). Patients were staged according to international Federation of Gynecology and Obstetrics (FIGO) criteria 2014 based on World Health Organization (WHO) guidelines. Histological subtype was confirmed by experienced gynecologic pathologists based on morphology, and when available P53 immunohistochemistry staining. The presence of tumor tissue was confirmed on H&E slides and representative locations with tumor tissue were selected for the TMA. OS was calculated from the date of initial treatment (either primary surgery or first cycle of neo-adjuvant chemotherapy) and was last updated in July 2020.

**Table 1:** Patient characteristics cohort N=176.

		N	%
FIGO stage			
	II	9	5
	III	133	76
	IV	34	19
	Unknown	0	0
BRCA status			
	Mutant	16	9
	Wildtype	50	28
	Unknown	110	63
Primary treatment			
	Primary surgery	83	47
	Neoajuvant chemotherapy	93	53
Surgery outcome			
	Macroscopic tumor	87	49
	No macroscopic tumor	87	49
	Unknown	2	1

FIGO, Fédération Internationale de Gynécologie et d’obstétrique.

### Immunohistochemical staining

For immunohistochemistry (IHC), tissue microarray (TMA) sections were constructed as described previously (36). Formalin-fixed, paraffin-embedded (FFPE) TMA slides were dewaxed in xylene and later rehydrated by using degraded concentrations of ethanol. Antigen retrieval was initiated (10 mM citrate buffer, pH6) and endogenous peroxidase activity was blocked (30% H_2_O_2_ solution). Slides were stained with rabbit anti-human CD103 mAb (anti-αEβ7-integrin, Abcam, Cambridge, UK, 1:200) before incubation overnight at 4 °C. The next day, slides were incubated with Envision-HRP anti-rabbit and later amplified with fluorophore cyanine 5 according to manufacturer’s instructions (TSA Cyanine 5 (Cy5) detection Kit, Perkin Elmer, 1:50). Next, the slides were stained with mouse anti-human CD8 (DAKO, Heverlee, Belgium, clone C8/144B, 1:50) before incubation overnight 4 °C. On the third day, slides were incubated with Envision-HRP anti mouse and amplified using the Fluorescein detection kit (Perkin Elmer, 753001KT, 1:50) according to the manufacturer’s instructions. Afterwards, the slides were stained with rabbit anti-TIM-3 mAb before incubation overnight at 4°C. The next day, the slides were incubated with Envision-HRP anti-rabbit and amplified by using TSA Cyanine 3 (Cy3) (Perkin Elmer, 753001KT, 1:50). The sections were embedded in prolong diamond anti-fade mounting medium with DAPI (Life Technologies).

### Image acquisition and analysis

Sections were scanned using a TissueFAXS imaging system (TissueGnostics, Vienna, Austria). Processed channels were merged using ImageJ. Within each core, single-positive CD8 cells, double-positive CD103/CD8 cells and triple-positive CD8/CD103/TIM-3 cells were counted, and the percentage of tumor/stromal surface was estimated. The slides were counted manually by 2 individuals who were blinded for the clinicopathological data. Afterwards, scores of the 2 individual counters were compared and differences in counts of over 10% were reanalyzed until consensus was reached.

### Ethics

The study was approved by the local ethics review board under Register number 201700448.

### Single cell mRNA sequencing data analysis

A single-cell tumor immune atlas based on over 500,000 cells from 217 patients and 13 cancer types (described in (34)) was utilized to evaluate gene expression in the tumor immune microenvironment (TME). The dataset was downloaded in the form of a RDS file containing the Seurat object. The data was uploaded into Seurat V4 in R language version 4.0.3. Within this cell atlas, immune cell fractions were pre-separated into 25 different clusters using canonical markers and curated gene signatures (B cells, proliferative B cells, plasma B cells, naive T cells, regulatory T cells, T helper cells, Th 17 cells, proliferative T cells, recently activated CD4-positive T cells, naive-memory CD4-positive T cells, transitional memory CD4-positive T cells, pre-exhausted CD8-positive T cells, cytotoxic CD8-positive T cells, effector memory CD8-positive T cells, terminally exhausted CD8-positive T cells, NK cells, secreted phosphoprotein 1 (SPP1) tumor-associated macrophages (TAMs), M2 TAMs, pro-inflammatory TAMs, proliferative monocytes and macrophages, monocytes, conventional dendritic cells (cDC), plasmacytoid dendritic cells (pDC), myeloid DC (mDC) and mast cells). Most cells were negative for TIM-3 and therefore cells with non-zero TIM-3 expression were considered TIM-3-positive. Differential expression was calculated by using the FindMarkers function from Seurat with MAST as the method of choice (37).

### TIL flow cytometric analysis

Tumor infiltrating lymphocyte (TIL) extraction was performed on ovarian cancer tissue obtained during surgery collected in the University Medical Center Groningen, The Netherlands. This study was carried out in the Netherlands in accordance with International Ethical and Professional Guidelines (the Declaration of Helsinki and the International Conference on Harmonization Guidelines for Good Clinical Practice. The use of anonymous rest material is regulated under the code for good clinical practice in the Netherlands and had been processed anonymously (38). Patients had given consent to use surgical material for research purposes. Primary patient TILs used for analysis of the TIL phenotype were isolated from fresh tumor samples obtained during cytoreductive surgery. Thawed TILs were resuspended in FACS tubes in a final volume of 200 µl and stimulated with Cell Stimulation Cocktail (Thermo Fisher) for 12-16h at 37°C. Golgiplug (BD Bioscences) was added the last 4h of the culture. CD3, CD8, CD103, TIM-3 and CXCL13 expression was determined by mAbs specific for the corresponding human molecules conjugated with BV785 (CD3, Biolegend), BV421 (CD8, BD Bioscences), Fluorescein Isothiocyanate (CD103, BD Bioscences), Pe-CF594 (TIM-3, BD Biosciences) and APC (CXCL13, Invitrogen). Fix and Perm solutions A and B from Nordic MuBio (Susteren, the Netherlands) were used for analysis of intracellular molecules. Acquisition was done on a CytoFLEX flow cytometer (Beckman Coulter), and analysis was performed using FlowJo V10.5.3.

### Statistical analysis

Statistical analyses were performed using IBM SPSS Statistics for Windows, version 23 (IBM Corp., Armonk, N.Y., USA) and R (version 3.6.2). Immune cell densities were log2 transformed for analysis. Clustering of cases was done by hierarchical clustering using Ward’s minimum variance method in R using package heatmap. Correlations between immune clusters and clinical and histopathological variables were analyzed using Multiple regression analysis in SPSS. Analysis of OS as a function of immune cell density was performed in R using package Survminer. Survival curves were plotted using the Kaplan–Meier method. A p-value of <0.05 was used as cutoff for significance. Patients were divided into high or low/no infiltration clusters, determined based on the optimal cut-off.

## Results

### Patterns of T cell infiltrates in EOC patients

**Figure 1:**
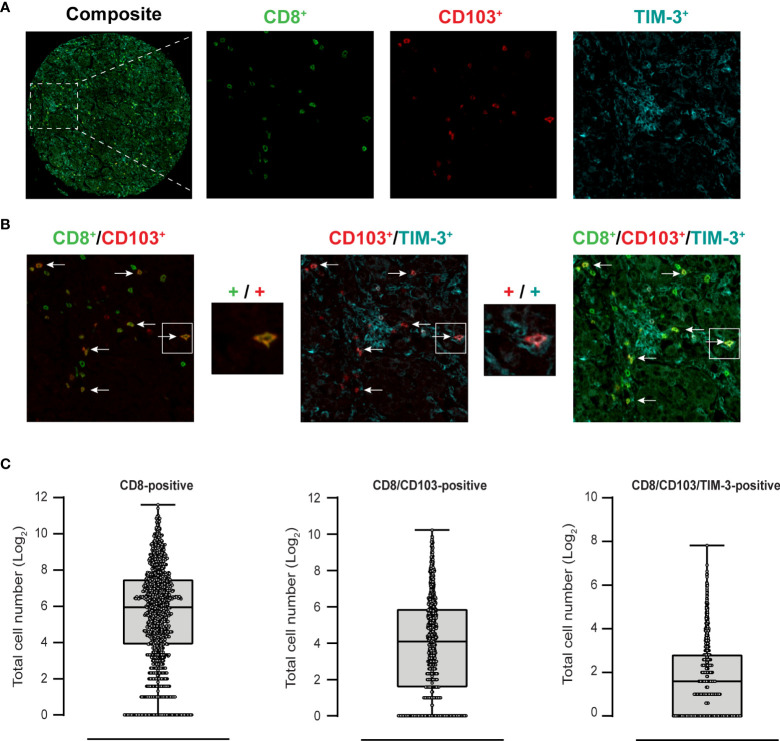
Tumor infiltration of immune cell subsets CD8-positive, CD8/CD103-positive and CD8/CD103/TIM-3-positive in EOC core samples. Exemplary processed IHC slide stained with antibodies targeting CD8 (green), CD103 (red) and TIM-3 (cyan) and corresponding secondary antibodies **(A)**. TMA sections were scanned using a TissueFAXS imaging system. Processed channels were merged using ImageJ. Fluorescent overlay analysis revealing CD8/CD103 double-positive, CD103/TIM-3 double positive and CD8/CD103/TIM-3 triple-positive cells **(B)**. Single-, double- and triple-positive cell populations were counted using ImageJ. Total raw cell counts of the single-, double- and triple-positive cell populations are Log2 transformed and displayed in **(C)**.

**Figure 2:**
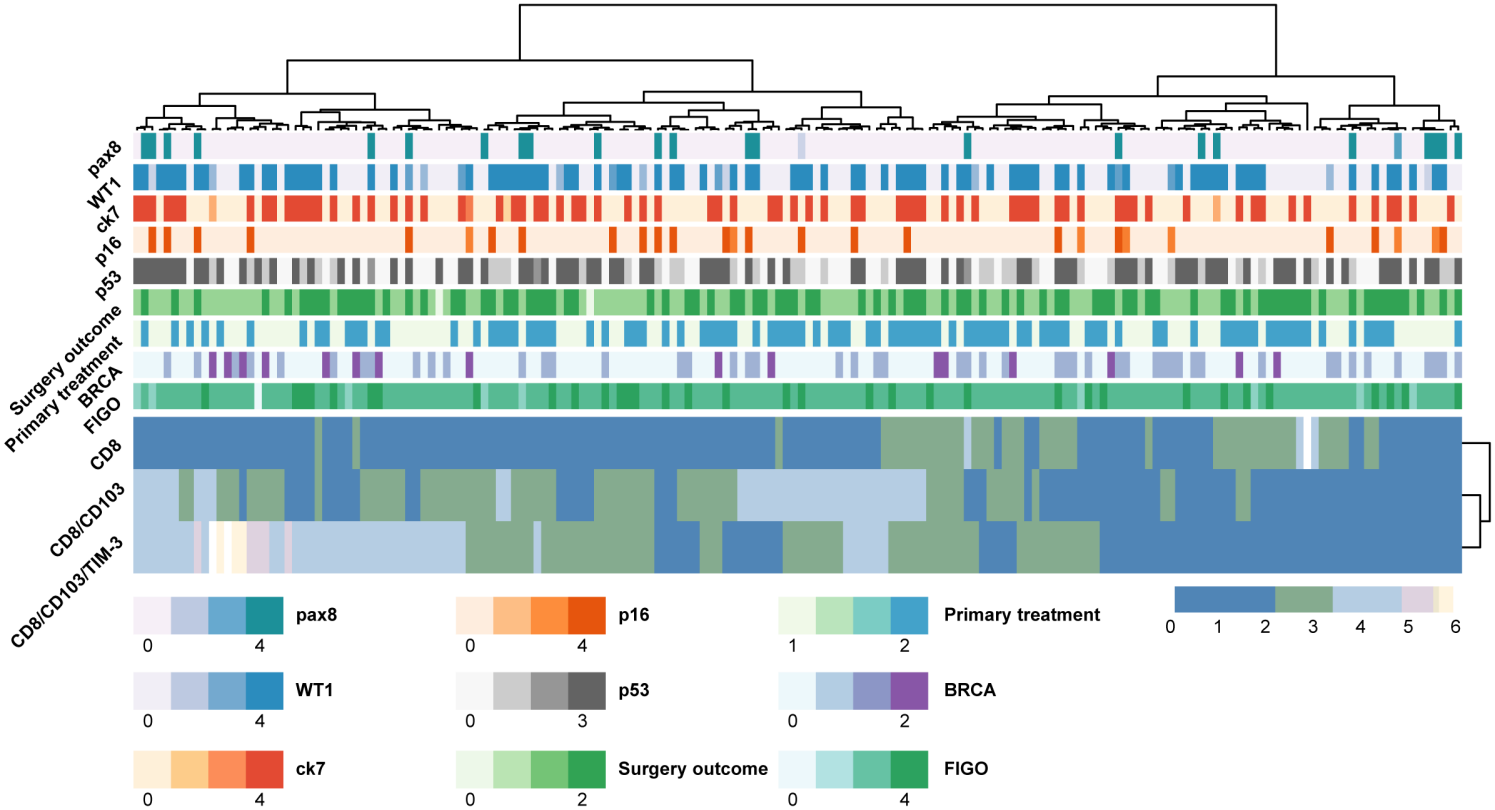
Patterns of infiltration of CD8/CD103/TIM-3-expressing immune cell subsets. Heatmap displaying infiltration of CD8/CD103/TIM-3-expressing immune cell subsets in EOC core samples. Hierarchical cluster analysis of all samples displayed three clusters based on immune cell populations: CD8-positive, CD8/CD103-positive and CD8/CD103/TIM-3-positive. Clinical characteristics are displayed for each sample, including FIGO-stage, BRCA-status, primary treatment strategy (primary debulking surgery (PDS) versus neoadjuvant chemotherapy (NACT)) and the presence of macroscopic disease after surgery (complete versus incomplete). Histopathological markers determined during diagnostic workup including p53, PAX8, WT1 and CK7 are further displayed. Clustering of cases was done by hierarchical clustering using Ward’s minimum variance method in R using package pheatmap. Correlations between immune clusters and clinical and histopathological variables were analyzed using multiple regression analysis in SPSS.

Immunofluorescent analysis of EOC patient tissue identified CD8-positive, CD103-positive, TIM-3-positive cells (**Figure 1A**) and all combinations thereof (**Figure 1B**). TIM-3-positive cells represented a relatively small subpopulation, with median cell counts of 63, 16 and 2 for CD8 single-positive (left), CD8/CD103 double-positive (middle) and CD8/CD103/TIM-3 triple-positive (right) populations, respectively (**Figure 1C**). Hierarchical clustering revealed that patient samples that displayed high infiltration levels of triple-positive cells were also characterized by infiltrates of double-positive cells (CD8/CD103 vs CD8/CD103/TIM-3 bars in **Figure 2**). Upon multiple regression analysis no significant association of the FIGO-stage, BRCA-status, primary treatment strategy or surgery outcome with any of the clusters was established (**Figure 2**). Interestingly, although statistical significance was not reached, an association between BRCA-status (purple bar) and the triple-positive CD8/CD103/TIM-3 cluster was identified. Further, multiple regression analysis of histopathological markers PAX8, WT1, CK7, P16 and P53 determined during diagnostic workup revealed no association with any of the immune clusters.

### TIL TIM-3 expression associates with improved survival in EOC

**Figure 3:**
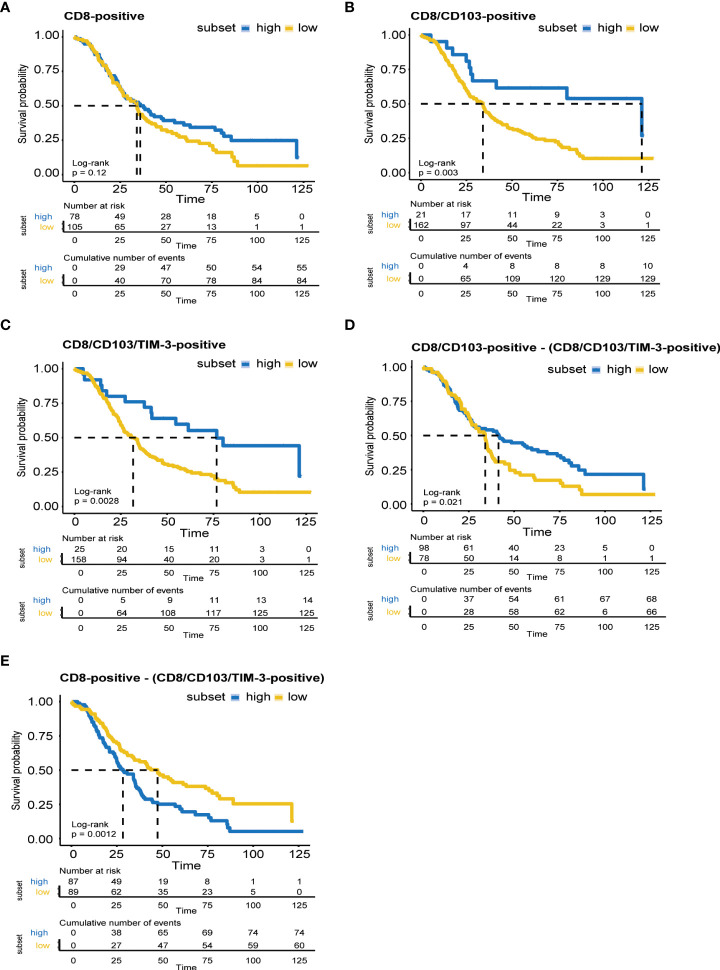
TIL TIM-3 expression associates with improved survival in EOC. Analyses of OS in months as a function of immune cell density based on immune cell populations CD8-positive **(A)**, CD8/CD103-positive **(B)**, CD8/CD103/TIM-3-positive **(C)**, CD8/CD103-positive - (CD8/CD103/TIM-3-positive) **(D)** and CD8-positive - (CD8/CD103/TIM-3-positive) **(E)** performed by Cox proportional hazard models in R using packages RMS and survival, plotted using package ggPlot2. Proportionality of hazards was confirmed by scaled Schoenfeld residuals. Optimal cutoff analysis was determined in R using package Survminer. Survival curves were plotted in R using Survminer by using the Kaplan–Meier method. A p-value of <0.05 was used as cutoff for significance.

Infiltration of CD8-positive T cells did not significantly associate with survival in the dichotomized patient cohort (**Figure 3A**, p = 0.12). In line with previous data, CD8/CD103-positive T cell infiltration did associate with a significant improvement in survival (**Figure 3B**, p = 0. 003). Importantly, although the total numbers of CD8/CD103/TIM-3 triple-positive infiltrated cells were much lower than that of single- and double-positive populations (see **Figure 1B**), the presence of triple-positive T cells also associated with a significantly better survival (**Figure 3C**, p = 0. 0028).

As the CD8/CD103 double-positive counts also include the CD8/CD103/TIM-3 triple-positive counts, an analysis was performed in which the triple-positive counts were removed from the double-positive counts, yielding a double – triple cluster (CD8/CD103-positive cells without (co-)expressing TIM-3) (**Figure 3D**). Interestingly, even though the double – triple population still significantly associates with (p = 0.021), the survival difference between the high and the low fraction was greatly reduced compared to the original, triple-positive high vs low cluster (**Figure 3B** vs **3D**). Of note, a CD8 single-positive population without cells (co-)expressing CD103 and TIM-3 was even associated with reduced survival (p = 0.0012, **Figures 3A** vs **3E**). Together, a clear survival benefit was detected in patients with high CD8/CD103/TIM-3 triple-positive tumor infiltration. Further, the higher survival probability observed for the single-positive high and double-positive high clusters can mainly be attributed to triple-positive cell also present within this population.

### Tumor-infiltrating terminally exhausted CD8-positive T cells have tumor-reactive signatures and co-express CXCL13 and TIM-3

**Figure 4:**
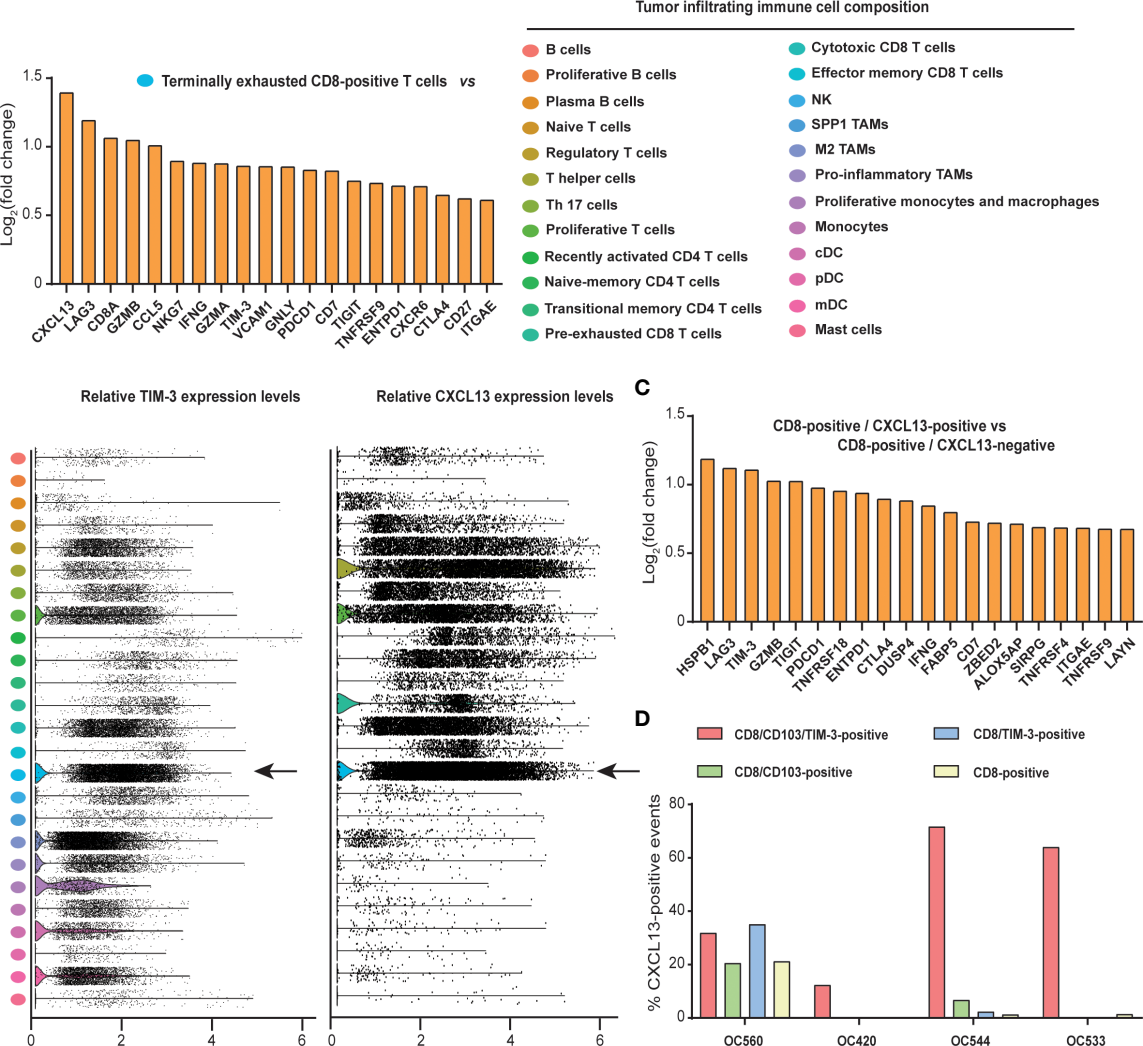
Tumor infiltrating terminally exhausted CD8-positive T cells have tumor-reactive signatures and co-express CXCL13 and TIM-3. Single-cell tumor immune atlas RNA sequencing data-set based on over 500,000 cells from 217 patients and 13 cancer types to evaluate gene expression in the tumor immune microenvironment. Immune cell fractions were pre-separated into 25 different clusters using canonical markers and curated gene signatures (**A**, right). Evaluation of DEGs within the terminally exhausted CD8-positive TIL cluster versus all the other tumor infiltrating immune cell clusters (**A**, left). Relative TIM-3 and CXCL13 expression found across the different immune cell fractions **(B)**. DEGs found within the CD8-positive CXCL13-positive vs CD8-positive CXCL13-negative analysis **(C)**. **(D)** % CXCL13 events found in CD8/CD103/TIM-3-positive (red bars), CD8/CD103-positive (green bars), CD8/TIM-3-positive (blue bars) and CD8-positive (yellow bars) populations from 4 different primary ovarian cancer patient TIL samples evaluated by flow cytometry.

To understand the observed differential survival, we analyzed differentially expressed genes (DEGs) within the pre-defined (34) terminally exhausted CD8-positive TIL cluster versus all the other tumor infiltrating immune cell clusters. This analysis revealed co-expression of several genes associated with exhaustion, such as Lymphocyte Activating 3 (LAG3), TIM-3, T Cell Immunoreceptor With Ig And ITIM Domains (TIGIT), Programmed cell death protein 1 (PDCD1), CD39 (encoded by the ENTPD1 gene) and Cytotoxic T Lymphocyte Associated Protein 4 (CTLA4) (**Figure 4A**, left). Genes associated with cytotoxicity, such as Granzyme B (GZMB), Natural Killer Cell Granule Protein 7 (NKG7), Interferon Gamma (IFNG), Granzyme A (GZMA), Granulysin (GNLY), TNF Receptor Superfamily Member 9 (TNFRSF9/4-1BB) and CD27 were likewise upregulated (**Figure 4A**, left).

In line with expectation, upregulated expression of tissue-resident memory T cell marker CD103 (encoded by the ITGAE gene) was detected, as well as upregulation of the B cell recruiting chemokine CXCL13 (**Figure 4A**, left). CXCL13/CD103/CD8 triple-positive TILs have previously been identified with B cell recruitment, TLS formation, neo-antigen burden and cytolytic gene signatures in human tumors and TIM-3 expression has been identified on CD39/CD103 double-positive tumor-reactive CD8-positive T cells (17, 25). Therefore, a possible TIM-3 and CXCL13 co-expression pattern within the tumor infiltrating immune-repertoire was further evaluated by us.

Both TIM-3 and CXCL13 expression was found across a range of cell types, with relatively high expression of both molecules found within the terminally exhausted CD8-positive T cell fraction (**Figure 4B**, see arrow’s). As expected, high CXCL13 expression was also found in T follicular helper cells (39). When comparing DEGs within the CXCL13-positive and negative fractions from the CD8-positve TIL subset, again exhausted and cytotoxic signatures were found (e.g., upregulation of LAG3, PDCD1, CTLA4, TNFRSF18 (GITR), IFNG, TNFRSF4 (OX40) and TNFRSF9 (4-1BB)) (**Figure 4C**). Interestingly, in this analysis upregulated expression of TIM-3 was also detected (**Figure 4C**, third bar from left), suggesting a possible co-expression profile with CXCL13. Confirmatory flowcytometric evaluation of CXCL13 expression on isolated EOC TILs revealed that CXCL13 was predominantly found within the CD8/CD103/TIM-3 triple-positive fraction compared to it’s single- and double-positive counterparts (**Figure 4D**, red bars vs all others). A representative gating strategy is displayed in **Supplemental Figure 1**.

## Discussion

In the present study, we demonstrated that a small population of CD8/CD103/TIM-3 triple-positive TILs was present in the tumor micro-environment of EOC patients. This triple-positive population associated with improved survival in EOC. Additionally, by evaluating gene signatures in terminally exhausted CD8-positive TILs from various cancer types, an effector/exhaustive/tumor-reactive profile with a co-expression pattern of CD103, TIM-3 and CXCL13 was found.

The prognostic value of TIM-3 expression on TILs is a subject of debate, as for some cancers high expression of TIM-3 within the TIL population has been associated with poor prognosis, whereas in others it was found to have a positive impact on prognosis (reviewed in (40)). For example, even though PD-1/TIM-3 double-positive CD8-positive TILs in ovarian cancer displayed enhanced potential for cytokine production and proliferation compared to other CD8-positive TIL subsets, patients highly expressing PD-1 and TIM-3 in TILs had reduced progression free survival compared to patients with low PD-1 and TIM-3 TIL expression. However, no significant difference for overall survival was observed (21). Likewise, in oropharyngeal squamous cell carcinoma (OPSCC) TIM-3 expression in TILs was associated with a higher number of CD8-positive TILs, whereas no significant impact on overall survival was observed (41). In gastrointestinal stromal tumors (GIST) on the other hand, TIM-3 expression levels on TILs were an independent predictor of patients’ overall survival and disease-free survival (42). Interestingly, although PD-1 and TIM-3 expressing TILs in diffuse large B-cell lymphoma (DLBCL) displayed an exhausted phenotype, their actual total numbers were expanded, and they expressed high levels of cytotoxic molecules (43). Promisingly, their proliferative potential and cytokine release could subsequently be restored by PD-1 or TIM-3 blockade. Solely evaluating TIM-3 expression levels on total TIL population is therefore not sufficient to define the subset of TILs that associate with survival. For this reason, the identification of a more relevant TIL population, like reported here, in terms of association with patient survival is of interest.

In the current report, no significant association of the clinical characteristics FIGO-stage, BRCA-status, primary treatment strategy or surgery outcome with any of the evaluated immune cell clusters was established, although an association between BRCA-status and the CD8/CD103/TIM-3 triple-positive cluster was observed. A BRCA 1/2-status has previously been linked to immunogenicity and survival and might also be predictive for response to immune checkpoint inhibitors (44–46). Further, the survival benefit observed in the current report for EOC patients can mainly be attributed to the presence of CD8/CD103/TIM-3 triple-positive TILs, with limited to no impact of the CD8/CD103 double-positive or the CD8 single-positive cell population on survival. In accordance, in patients with clear cell renal cell carcinoma (ccRCC) it was shown that although extensive CD8-positive T cell infiltrate levels were observed, due to the absence of TLSs and expression of immune checkpoints there was an increased risk of disease progression (33). As in the current manuscript no evidence is provided that CXCL13 produced by CD8/CD103/TIM-3-triple positive cells leads to TLS formation, evaluation of additional immune checkpoints and assessing the presence of TLSs in the same EOC cohort as in the present study may further help implementing of our observations.

CXCL13 is a key molecular determinant of the formation of prognostically favorable TLSs and is considered to be a surrogate marker for tumor TLS (26). Multiple studies have linked the expression of CXCL13 to patient prognosis and its potential as response biomarker to immunotherapy (26, 31, 39, 47). CXCL13 plays an important role in shaping the anti-tumor microenvironment by facilitating immune cell recruitment, their activation and regulating the adaptive immune response (48). Interestingly, in our scRNAseq analysis, CXCL13 expression was found within the terminally exhausted CD8-positive T cell fraction next to that of TIM-3 and CD103. Expression of CXCL13 was subsequently also confirmed on the CD8/CD103/TIM-3 triple-positive fraction in primary EOC samples. Within the CD8 subsets, CD8/CD103/TIM-3-positive cells predominantly express CXCL13 and their infiltration is associated with improved patient survival in EOC. However, this finding may be limited to EOC as other studies show that CD8/CXCL13-positive cells are also associated with poor clinical outcomes and display an immunoevasive contexture in the TME of ccRCC and gastric cancer (49, 50).

Multiple DEGs, collectively reflecting an exhaustive phenotype with a tumor-reactive potential, were furthermore found in the scRNAseq analysis when comparing tumor exhaustive CD8-positive TILs to the complete tumor infiltrating immune cell repertoire. For example, upregulated expression of LAG3, TIM-3, TIGIT, PDCD1, CTLA4 confirmed a transcriptome associated with exhaustion (51, 52). Further, upregulated expression of CD39 (ENTPD1) was detected, a marker of persistent TCR stimulation on exhaustive T cells (17, 53), co-expression of which with tissue-resident memory T cell marker CD103 (ITGAE) has been identified on tumor-reactive CD8-positive T cells in human solid cancers (17). The anti-tumor CD103-positive CD8-positive T cell subset has furthermore been associated with chemokine CXCL13 expression (28), and is in line with the co-expression with TIM-3 in our analysis.

Targeting inhibitory receptors like TIM-3 to reverse T cell exhaustion is of potential therapeutic interest for a variety of cancers (54–56). In this respect, antagonistic antibodies targeting TIM-3 on tumor-specific exhausted T cells alone or in combination with PD-1 or PD-L1 targeting antibodies are under clinical evaluation (57–59). As monotherapy however, none to limited anti-tumor activity has been reported so far (57, 58). A bi-specific antibody targeting both TIM-3 and PD-L1 has also been clinically evaluated, but it’s further development was terminated due to unexpected immunogenicity upon targeting of both the TIM-3 and PD-L1 arms (60). Indeed, the tumor-specific role of T cell expressed TIM-3 as well as potential tumor cell-expressed TIM-3 will need to be clarified in order to rationally design TIM-3 targeted immunotherapy.

In conclusion, we identified a small set of CD8/CD103/TIM-3-expressing tumor infiltrated T cells in EOC patients associated with improved EOC patient survival. Therefore, CD8/CD103/TIM-3 triple-positive TILs may be a prognostic marker for EOC and represents a target population of interest for reactivation by immunotherapeutics. Further, DEG analysis revealed upregulated expression of co-stimulatory, cytotoxic, and exhaustive genes, and notably that of CXCL13, CD103 and TIM-3 within the terminally exhausted CD8-positive T cell fraction. Due to the observed co-expression pattern of TIM-3 and CXCL13, TIM-3 expression on CD8/CD103-double positive TILs may be used as surrogate marker for prognostically favorable CXCL13-positive CD8-positive TILs and may have prognostic value itself.

## Data availability statement

The original contributions presented in the study are included in the article/**Supplementary Material**. Further inquiries can be directed to the corresponding authors.

## Ethics statement

The study was approved by the local UMCG ethics review board under Register number 201700448. The patients/participants provided their written informed consent to participate in this study.

## Author contributions

Conceptualization: MV, VB, GH, HN, MB and EB. Data curation: MV, VB, JA, AP and MB. Formal analysis: MV, VB, JA, AP and MB. Investigation: MV, VB, MB and EB. Supervision: EB. Writing – original draft: MV and VB. Writing – review & editing: VM, MB and EB. All authors contributed to the article and approved the submitted version.

## Funding

Supported by a grant from the European Union (under the Marie Sklodowska-Curie grant agreement No 813871).

## Conflict of interest

The authors declare that the research was conducted in the absence of any commercial or financial relationships that could be construed as a potential conflict of interest.

## Publisher’s note

All claims expressed in this article are solely those of the authors and do not necessarily represent those of their affiliated organizations, or those of the publisher, the editors and the reviewers. Any product that may be evaluated in this article, or claim that may be made by its manufacturer, is not guaranteed or endorsed by the publisher.

## Supplementary material

Supplementary Figure 1 A representative gating strategy for flowcytometric evaluation of CXCL13 expression on isolated EOC TILs within the CD8/CD103/TIM-3 triple-positive fraction compared to it’s single- and double-positive counterparts. Cells were gated, followed by the selection of the viable CD3/CD8-positive subset. Here the CD8/CD103/TIM-3 subsets were evaluated for CXCL13 expression. An “all abs -CXCL13 antibody” was taken along as control.
