# Supplementary figures and images for: Tumor infiltrating CD8/CD103/TIM-3-expressing lymphocytes in epithelial ovarian cancer co-express CXCL13 and associate with improved survival
Source: Front Immunol. 2022 Oct 21;13:1031746. doi: 10.3389/fimmu.2022.1031746 (PMC9633842; doi:10.3389/fimmu.2022.1031746)

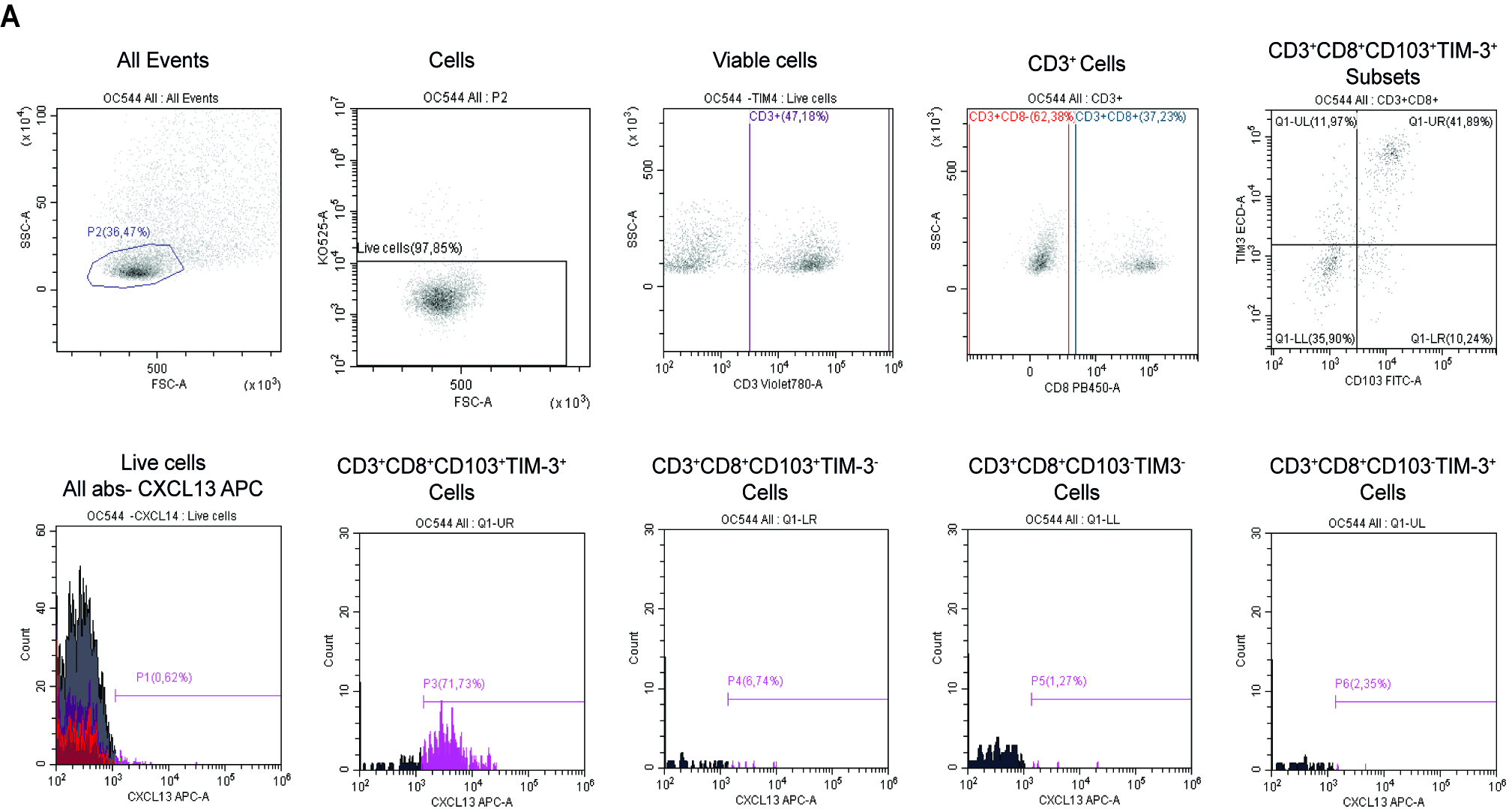

Supplement: Supplementary Figure 1 — A representative gating strategy for flowcytometric evaluation of CXCL13 expression on isolated EOC TILs within the CD8/CD103/TIM-3 triple-positive fraction compared to it’s single- and double-positive counterparts. Cells were gated, followed by the selection of the viable CD3/CD8-positive subset. Here the CD8/CD103/TIM-3 subsets were evaluated for CXCL13 expression. An “all abs -CXCL13 antibody” was taken along as control. [file Image_1.tif]
